# Psychometrics of the breastfeeding self-efficacy scale and short form: a systematic review

**DOI:** 10.1186/s12889-024-17805-6

**Published:** 2024-02-29

**Authors:** Cindy-Lee Dennis, Karen McQueen, Justine Dol, Hilary Brown, Cheryl Beck, Shefaly Shorey

**Affiliations:** 1 Lawrence S. Bloomberg Faculty of Nursing, University of Toronto, 155 College Street, Toronto, ON M5T 1P8 Canada; 2 Lakehead University, Thunder Bay, ON Canada; 3 IWK Health, Halifax, Canada; 4 University of Toronto, Scarborough, ON Canada; 5 University of Connecticut, Storrs, CT USA; 6 National University of Singapore, Singapore, Singapore

**Keywords:** Breastfeeding self-efficacy, Systematic review, Psychometric evaluation

## Abstract

**Background:**

The Breastfeeding Self-Efficacy Scale and its short-form were developed in Canada and have been used internationally among numerous maternal populations. However, the psychometric properties of the scales have not been reviewed to confirm their appropriateness in measuring breastfeeding self-efficacy in culturally diverse populations. The purpose of this research was to critically appraise and synthesize the psychometric properties of the scales via systematic review.

**Methods:**

The Preferred Reporting Items for Systematic Reviews and Meta-Analyses (PRISMA) guidelines were followed. Three databases (EMBASE, MEDLINE, and PsycINFO) were searched from 1999 (original publication of the Scale) until April 27, 2022. The search was updated on April 1, 2023. Studies that assessed the psychometric properties of the BSES or BSES-SF were included. Two researchers independently extracted data and completed the quality appraisals.

**Results:**

Forty-one studies evaluated the psychometrics of the BSES (*n* = 5 studies) or BSES-SF (*n* = 36 studies) among demographically or culturally diverse populations. All versions of the instrument demonstrated good reliability, with Cronbach's alphas ranging from .72 to .97. Construct validity was supported by statistically significant differences in mean scores among women with and without previous breastfeeding experience and by correlations between the scales and theoretically related constructs. Predictive validity was demonstrated by statistically significant lower scores among women who ultimately discontinued breastfeeding compared to those who did not.

**Conclusion:**

The BSES and BSES-SF appear to be valid and reliable measures of breastfeeding self-efficacy that may be used globally to (1) assess women who may be at risk of negative breastfeeding outcomes (e.g., initiation, duration and exclusivity), (2) individualize breastfeeding support, and (3) evaluate the effectiveness of breastfeeding interventions.

**Supplementary Information:**

The online version contains supplementary material available at 10.1186/s12889-024-17805-6.

## Article summary

The BSES and BSES-SF appear to be valid and reliable measures of breastfeeding self-efficacy that can be used globally to identify women at-risk for poor breastfeeding outcomes.

## Background

The benefits of breastfeeding for disease prevention and health promotion are undisputed. If breastfeeding exclusivity occurred at a near universal level among young infants, it is estimated that 823,000 deaths in children under the age of five could be prevented annually [1]. Due to the beneficial effects, exclusive breastfeeding for the first six months postpartum is recommended internationally. While the overall global rate of exclusive breastfeeding for infants less than six months of age is currently 44% [2], the World Health Organization has set a goal to achieve at least a 50% exclusivity rate by 2025 [3]. One potential highly effective strategy to improve exclusive breastfeeding rates is to tailor supportive resources among women at risk of poor breastfeeding outcomes [4]. Breastfeeding self-efficacy is one possible modifiable variable that has been consistently associated with positive breastfeeding outcomes, including exclusivity [5, 6].

Breastfeeding self-efficacy is defined as a mother’s confidence in ability to breastfeed [7] and predicts “whether a mother chooses to breastfeed, how much effort she will expend, whether she will persevere in her attempts until mastery is achieved, whether she will have self-enhancing or self-defeating thought patterns, and how she will emotionally respond to breastfeeding difficulties” (p. 736). Consistent with Bandura’s Social Learning Theory [8], Dennis’ breastfeeding self-efficacy theory [7] hypothesizes that maternal breastfeeding self-efficacy may be affected by four primary sources (e.g., antecedents) including [1] performance accomplishments (e.g., past breastfeeding experiences), [2] vicarious experiences (e.g., watching other women breastfeed), [3] verbal persuasion (e.g., encouragement from influential others such as friends, family, and lactation consultants), and [4] physiological responses (e.g., pain, fatigue, stress, depression, anxiety). Thus, an individual’s self-efficacy may be enhanced by altering the sources of information.

The 33 item five-point Likert Scale Breastfeeding Self-Efficacy Scale (BSES) was developed by Dennis [9]. Each item was preceded by the phrase I can always…, with responses ranging from not at all confident to always confident. A psychometric evaluation of the BSES were initially conducted with a sample of 130 Canadian women, resulting in a Cronbach's alpha of 0.96 and 73% of all item-total correlations falling within the 0.30-0.70 range [9]. Factor analysis revealed two distinct factors: (1) Breastfeeding Technique Subscale, and (2) Intrapersonal Thoughts Subscale. In the initial sample, BSES scores were predictive of breastfeeding duration at 6 weeks postpartum. Internal consistency data, however, suggested the presence of redundant items and so the scale was retested in a larger Canadian sample [10]. After a detailed item analysis, 19 items were deleted, culminating in the 14-item BSES-Short Form (SF) [10]. Total scores range from 14–70 with lower scores indicating lower breastfeeding self-efficacy. Today, the BSES-SF is widely used internationally to identify women who may be at-risk for prematurely discontinuing breastfeeding.

In a meta-analysis of 11 trials evaluating breastfeeding self-efficacy interventions among women of term infants, researchers found that intervention groups participants were 1.56 times more likely to be breastfeeding at 1 month increasing to 1.66 times more likely at 2 months postpartum [5]. The researchers concluded that interventions that began in the postpartum period that used combined delivery settings (e.g., hospital and community) or were theoretically derived, had the largest effect on breastfeeding self-efficacy and rates of breastfeeding. Further, meta-regression analysis suggested the odds of exclusive breastfeeding increased by 10% among intervention participants for each 1-point increase in mean BSES scores between the intervention and control groups. Similarly, another systematic review and meta-analysis of 12 trials [11] revealed that women receiving breastfeeding support interventions had significantly improved breastfeeding self-efficacy scores during the first 4 to 6 weeks postpartum (*SMD* = 0.40, *p* = 0.006, 95% CI [0.11, 0.69]) and decreased perceptions of insufficient milk supply (median, 3.3, *p* < 0.001).

While a general review of the BSES-SF was completed [12], no systematic review has been undertaken to examine the application of both the BSES and BSES-SF in culturally and demographically diverse populations. The cross-cultural adaptation of the scale, as well as the validation among mothers with specific demographic or clinical characteristics, is crucial for enabling the instrument to serve as a useful international measure of breastfeeding self-efficacy. Furthermore, the development of modified versions of the instrument with sound psychometric properties facilitates an awareness of—and sensitivity to—the needs and perceptions of mothers with varied cultural, socioeconomic, and medical histories. Thus, the objective of this review was to appraise the validated BSES and BSES-SF in culturally and demographically diverse contexts.

## Methods

### Design

We performed a systematic review of quantitative studies using the Preferred Reporting Items for Systematic Reviews and Meta-Analyses (PRISMA) guidelines [13]. A protocol was not registered.

### Sample: Defining the articles reviewed

**Fig.1:**
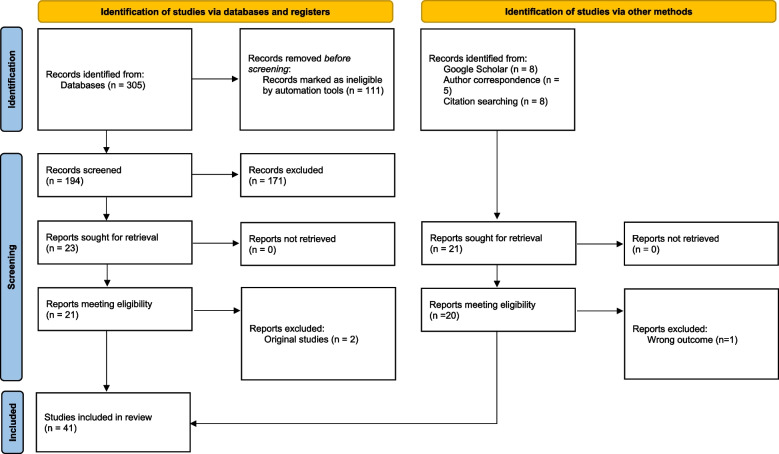
PRISMA Flow diagram

Quantitative studies (e.g., methodological, cross-sectional, cohort) were included if they used the BSES or BSES-SF and provided data concerning both the reliability and validity of the scale. Studies that aimed to determine predictive cut-off scores for the scale were also included if they analyzed the sensitivity and specificity of the scale at the identified cut-off. A total of 41 studies were included in the review. All studies were published in English; thus, there were no exclusions for language. Figure 1 displays a flowchart of the search strategy and the study selection process.

### Data collection: The search strategy and process

The initial search was conducted for published studies between 1999 (the publication year of the original BSES) and December 2021. Additional studies were identified via reference list searches and doing a ‘cited by’ search on Google Scholar and Medline as of April 1, 2023. We also contacted experts in the field to retrieve any data from recently completed studies not yet published. Searches of the literature were conducted using EMBASE, MEDLINE, and PsycINFO. The term “Breastfeeding Self-Efficacy Scale” was searched and Medical Subject Heading (MeSH) terms for “breastfeeding”, “self-efficacy”, “cultural adaptation”, “psychometric”, and “validated” were broadened to capture relevant literature. The search strategy is presented in Table S1.

### Measurement

A structured data extraction form was developed to organize data from the studies by publication year, study location, objectives, population, validation of the BSES or BSES-SF, language used for the scale, time of administering the scale, and means and standard deviations (SD) of BSES or BSES-SF scores. Study quality was assessed using criteria suggested by Shrestha et al. [14] and Mirza and Jenkins [15] and included: (1) clarity of the study aim, (2) sample size justification, (3) sample representativeness, (4) clear inclusion and exclusion criteria, (5) description of maternal demographic data, (6) reporting of a response rate, (7) appropriate statistical analyses, and (8) evidence of participant informed consent. Possible scores of 1 (e.g., sample size justified) or 0 (e.g., sample size not justified) were used and combined to give a possible total score of 8 for each study. Quality criteria specific to the translation and validation of psychometric tools such as the BSES and the BSES-SF were adapted from Shrestha et al.’s [14] additional quality assessment parameters, which included an assessment of the translation methods used, cultural adaptation, as well as any modifications made to items in the scale. Two authors independently extracted data and completed the quality appraisals. When information was unclear authors reviewed the data and/or had a third author review to achieve consensus.

### Data analysis

Data pertaining to the psychometrics of the scales were summarized and included internal consistency, factor analyses, known groups analyses, and predictive validity of the scales, as well as the correlation of the scores with other theoretically related constructs, negative and positive predictive value, and sensitivity and specificity. As no other measure of breastfeeding self-efficacy was used in these studies for comparison, instruments to which BSES or BSES-SF scores were compared differed slightly among the analyzed studies. Additional scales administered in several studies included the Edinburgh Postnatal Depression Scale (EPDS) [16] and the General Self-Efficacy Scale (GSES) [17]. Since self-efficacy has been shown to be negatively influenced by psychological disorders such as depression; a negative correlation between BSES and EPDS scores was hypothesized. Based on the rationale that breastfeeding self-efficacy should be enhanced among mothers with higher overall self-efficacy, a positive correlation between BSES and GSES scores was also hypothesized.

## Results

### Characteristics of included studies

The search yielded 305 studies, with 194 screened after duplicates were removed through automation. Of these screened studies, 171 did not meet the inclusion criteria, leaving 21 eligible studies after Dennis’ two original BSES [9] and BSES-SF [10] studies were excluded. Sixteen additional studies were identified via reference searches and reverse Google Scholar searches and five studies were identified pre-publication through author correspondence, with one not meeting the inclusion criteria. In total, 41 studies were included in the review with five focused on the original BSES and 36 presenting data on the BSES-SF. Of the BSES-SF studies, three reported on a modified scales for fathers [18–20] and three reported on a modified scale for mothers of preterm infants [21–23]. All included studies were of high quality with scores ranging from four to 8 eight (see Table S2).

Characteristics of the included studies are presented in Table 1. For the five studies validating the BSES, sample sizes ranged from 100 to 276 participants, with 60% falling within the recommended sample size for psychometric assessments of 5–10 subjects per item (Nunnally and Bernstein: Psychometric theory, unpublished). The 36 studies assessing the BSES-SF had sample sizes ranging from 18–1,524, all except two [24, 25] surpassing the minimum recommended sample size. Participants were primarily recruited from maternity wards of urban hospitals and most sample characteristics were representative of the regional population. The most common time to assess breastfeeding self-efficacy was in-hospital during the postpartum period (*n* = 17; 41%). However, the timing of assessment varied among some studies with assessment of breastfeeding self-efficacy conducted during the third trimester of pregnancy [26, 27], postnatally (time not reported) [28, 29], at 1 week [27], or open time periods such as two to six weeks [20].

**Table 1:** Language of validation, objective, sample population and mean BSES/BSES-SF scores for all included studies

Author (Year)	Study Aim	City/Region, Country	Study Population/Sample Size	Scale Language	Time of BSES Assessment	Mean BSES / BSES-SF Scores (SD)	^a^Quality
**Breastfeeding Self-Efficacy Scale (BSES)**							
Creedy 2003 [26]	To psychometrically test the BSES antenatally and at 1 and 16 weeks postpartum	Brisbane, Australia	276 women at 36 + weeks gestation recruited from a university hospital	English	Antenatal: 3rd trimester Postnatal: 1 and 16 weeks	Antenatal: 126.16 (23.85) 1 week postnatal: 139.86 (23.87) 16 weeks postnatal: 142.26 (21.25)	8
Dai 2003 [30]	To translate the BSES into Mandarin and determine its psychometric properties	Tianjin, China	186 women 37 + weeks gestation recruited from an obstetric hospital	Mandarin Chinese	Postnatal: In-hospital	118.78 (16.53)	6
Eksioglu 2011 [31]	To translate the BSES into Turkish and assess its psychometric properties	Altındağ, Izmir, Turkey	165 women 37 + weeks gestation recruited from two mother and child health-care units	Turkish	Postnatal: 1, 4 and 8 weeks	1 week: 151.22 (12.39) 4 weeks: 154.99 (11.51) 8 weeks: 155.52 (11.35)	6
Molina Torres 2003 [32]	To translate the BSES into Spanish and determine its psychometric properties	San Juan, Puerto Rico	100 women 37 + weeks gestation recruited from a private hospital	Spanish	Postnatal: In-hospital	131.8 (22.07)	6
Oriá 2009 [33]	To translate and psychometrically assess the BSES among women living in Fortaleza, Brazil	Fortaleza, Brazil	117 women 30 + weeks gestation recruited during a prenatal visit at a teaching hospital	Portuguese	Antenatal: third trimester	NR	5
**Breastfeeding Self-Efficacy Scale- Short Form (BSES-SF)**							
Amini 2019 [28]	To evaluate the reliability and validity of the BSES-SF among Iranian mothers	Tehran, Iran	379 mothers recruited from a health centre	Persian	Postnatal: NR	50.80 (8.91)	6
Asgarian 2020 [34]	To explore the validity and reliability of BSES-SF among Iranian Farsi-speaking mothers	Qom, Iran	174 women recruited from teaching hospital	Iranian Farsi	Postnatal: In-hospital	54.32 (10.5)	8
Balaguer- Martinez 2022 [35]	To assesses the relationship between the BSES-SF score and the risk BF cessation and determine the cut-off point in the scale	Spain	1845 mothers with an infant born at term (> 37 weeks) part of the LAyDI cohort	Spanish	Postnatal: within 15 days post birth, plus 1-, 2-, 4-, and 6-months post birth	59.76 (9.95)	7
Basu 2020 [36]	To translate into Hindi and to psychometrically test the BSES-SF	North-East Delhi, India	210 women with a child under 1 year recruited from well-baby and immunization clinics at health centers	Hindi	Postnatal: timing not reported	54.7 (16.1)	7
Boateng 2019 [37]	To adapt the BSES-SF to emphasize EBF for use in a setting where continued, but not exclusive breastfeeding is common; to conduct psychometric testing of this adapted scale	Gulu, Uganda	239 women recruited between 10–26 weeks gestation from a regional hospital	Acholi and Langi	Postnatal: 4 and 12 weeks	4 weeks: Cognitive Subscale: 13.5 (4.1) Functional Subscale: 17.2 (4.3) 12 weeks: Cognitive Subscale: 13.6 (4.1) Functional Subscale: 17.7 (4.3)	6
Brandão 2018 [38]	To examine the psychometric characteristics of an antenatal version of the BSES-SF among pregnant Portuguese women	Northern Portugal	373 women between 30 and 34 gestational weeks, recruited from two public hospitals	Portuguese	Antenatal: 3rd trimester	57.93 (7.90)	6
Chipojola 2022 [18]	To examine the psychometric properties of the paternal BSES-SF in Malawian fathers	Lilongwe, Malawi	180 fathers recruited from a hospital	Chichewa	Postnatal: In-hospital	50.2 (11.9)	7
Dennis 2011 [39]	To psychometrically assess the BSES-SF antenatally and postnatally among adolescents	Winnipeg, Canada	100 adolescents (15–19 years) 37 + weeks gestation recruited from two prenatal clinics at a teaching hospital	English	Antenatal: 3rd trimester (34 weeks) Postnatal: 1 and 4 weeks	Antenatal: 51.72 (7.69) 1 week postnatal: 56.23 (12.27)	6
Dennis 2018 [19]	To assess the psychometric properties of the BSES–SF among fathers	Toronto, Canada	214 fathers recruited from a postpartum unit in a large teaching hospital	English	Postnatal: In-hospital and 6 weeks	In-hospital: 48.96 (NR) 6 weeks: 54.54 (NR)	6
Dodt 2012 [40]	To psychometrically assess the BSES-SF among women living in northeast-Brazil	Fortaleza-CE, Brazil	294 low-income women were recruited from a teaching university hospital	Brazilian Portuguese	Postnatal: In-hospital	NR	6
Dos Santos 2016 [41]	To verify the reliability and validity of the BSES-SF in Brazilian adolescent mothers	Brazil	79 adolescent (13–19 years) mothers were recruited from a public maternity institution	Brazilian Portuguese	Postnatal: In-hospital	56.58 (6.11)	7
Gerhardsson 2014 [42]	To translate and psychometrically test the Swedish version of the BSES-SF	Uppsala, Sweden	120 women 37 + weeks gestation recruited at routine follow-up visits at the postnatal unit in a university hospital	Swedish	Postnatal: 1 week	57.4 (8.8)	7
Gregory 2008 [43]	To psychometrically assess the BSES-SF among a multicultural sample of mothers living in the United Kingdom	Birmingham, United Kingdom	165 women 36 + weeks gestation recruited from the maternity ward in a hospital	English	Postnatal: in-hospital and 4 weeks	In-hospital: 46.46 (12.75)	7
Handayani 2013 [24]	NR	Yogyakarta, Indonesia	18 women recruited with a child between 0–6 months	Indonesian	Postnatal: timing not reported	NR	6
Husin 2017 [27]	To translate and assess psychometrics of the Malay version of BSES-SF in both antenatal and postnatal mothers	Malaysia	101 antenatal women 32 + weeks gestation recruited from maternal and child health clinic and 104 postnatal women 37 + weeks gestation recruited at first postnatal home visit	Malay	Antenatal: 3rd trimester Postnatal: 1 week	Antenatal: 56.20 (8.75) Postnatal: 58.97 (8.68)	7
Iliadou 2020 [44]	To conduct psychometric testing of the Greek version of BSES-SF	Athens, Greece	173 women 32 + weeks gestation recruited from an outpatient maternity department at a hospital	Greek	Antenatal: 3rd trimester Postnatal: 3 days	Antenatal: 44.2 (11.1) Postnatal: 47.7 (12.1)	8
Ip 2012 [45]	To translate the BSES-SF into Chinese and to examine its psychometric properties	Hong Kong	185 women 37 + weeks gestation recruited from a hospital postpartum unit	Hong Kong Chinese	Postnatal: In-hospital, 4 and 24 weeks	In-hospital: 41.1 (10.7)	7
Ip 2016 [46]	To establish the construct validity and prognostic ability of the Mandarin version of BSES-SF	Guangzhou, China	562 women 37 + weeks gestation recruited from a teaching hospital	Chinese Mandarin	Postnatal: In-hospital	In-hospital: 47.3 (10.5)	6
﻿ Küçükoğlu 2023 [20]	To translate and psychometrically test the PBSES-SF among Turkish fathers	Konya, Turkey	221 fathers with an infant aged 2–6 weeks recruited from pediatric outpatient clinics at 2 research hospitals	Turkish	Postnatal: 2–6 weeks	47.32 (NR)	6
Maurer (n.d.) (Maurer, et al.: The breastfeeding self-efficacy scale - short form (BSES): German translation and psychometric assessment, unpublished)	To translate the BSES-SF into German and assess its psychometric properties	Germany and Austria	355 postpartum mothers via social media	German	Postnatal: up to 12 weeks	58.46 (8.42)	7
McCarter-Spaulding 2010 [47]	To assess the psychometric properties of the BSES-SF in Black women in the United States	Northeastern United States	153 women 37 + weeks gestation recruited from an urban teaching hospital	English	Postnatal: 1 and 4 weeks	Mean BSES-SF not reported	7
McQueen 2013 [48]	To test the reliability and validity of the BSES-SF among Aboriginal women	Ontario, Canada	130 Aboriginal women recruited from an urban tertiary care hospital or a rural community hospital	English	Postnatal: In-hospital	In-hospital: 51.32 (11.74)	8
Mituki 2017 [25]	To translate, validate and adapt the BSES‐SF tool in a resource restricted urban setting in Kenya	Kenya	42 women 37 + weeks gestation recruited from prenatal clinic at a hospital	Kiswahili	NR	60.95 (10.36)	6
Oliver-Roig 2012 [49]	To translate the BSES-SF into Spanish and assess its psychometric properties	Orihuela, Spain	135 women 36 + week gestation recruited from a public hospital	Spanish	Postnatal: In-hospital	51.94 (11.22)	6
Otsuka 2008 [50]	To examine the relationship between maternal perceptions of insufficient milk and breastfeeding confidence using the BSES	Tokyo and Kusatsu, Japan	262 women 37 + weeks gestation recruited from 2 obstetric hospitals	Japanese	Postnatal: In-hospital, 4-weeks	In-hospital: 44.7 (11.9) 4-weeks: 43.8 (12.3)	6
Pavicic-Bosnjak 2012 [51]	To translate and psychometrically assess the Croatian version of the BSES-SF	Zagreb, Croatia	190 women recruited from a teaching hospital	Croatian	Postnatal: In-hospital	55 (7)	6
Petrozzi 2016 [52]	To translate the BSES-SF into Italian and investigate its predictive ability	Lido di Camaiore, Italy	122 women recruited from a hospital	Italian	Postnatal: In-hospital	54.8 (9.4)	4
Radwan 2022 [53]	To translate and examine the psychometric properties of the BSES-SF among mothers in the United Arab Emirates	Dubai, Sharjah, Abu Dhabi, and Fujairah, United Arabic Emirates	457 women recruited from postpartum ward in 10 hospitals	Arabic	Postnatal: In-hospital	55.22 (11.93)	8
Sandhi 2022 [29]	To assess the psychometric properties of the BSES-SF among Indonesian mothers	Yogyakarta City, Indonesia	237 mothers 37 + weeks gestation recruited from five public health centers	Bahasa Indonesia	Postnatal: timing not reported	56.4 (7.2)	6
Tokat 2010 [54]	To translate and psychometrically assess the BSES-SF among pregnant and postpartum women in Turkey	Izmir, Turkey	144 pregnant women recruited during a regular antenatal visit at two private and two public outpatient clinics, and 150 women recruited from the postpartum ward at two public and one private hospital	Turkish	Antenatal: during their antenatal visit in the third trimester Postnatal: In-hospital	Antenatal: 58.52 (8.8) Postnatal: 60.09 (8.2)	8
Tokat 2020 [21]	To psychometrically assess the Turkish version of the modified BSES-SF among mothers of preterm infants	Izmir, Turkey	135 mothers < 37 weeks’ gestation were recruited from a NICU	Turkish	Postnatal: 1 week	43.32 (5.76)	7
Wheeler 2013 [22]	To psychometrically assess the modified BSES-SF among mothers of ill or preterm infants	Winnipeg, Canada	144 women of ill or preterm infants recruited from 2 hospitals	English	Postnatal: 1 week post hospital discharge	79.39 (9.98)	5
Witten 2020 [55]	To translate and psychometrically test the reliability and validity of the BSES-SF in the context of South Africa	Northwest province, South Africa	180 mothers with an infant < 2 weeks were recruited from 8 primary health care clinics	Setswana	Postnatal: < 2 weeks	Median = 66 (IQR 62–68)	8
Wutke 2007 [56]	To translate the BSES-SF into Polish and assess its psychometric properties	Lodz, Poland	105 women 37 + weeks gestation recruited from postpartum units in 5 hospitals	Polish	Postnatal: In-hospital	55.5 (8.4)	8
Yang 2020 [23]	To translate, transculturally adapt and assess the psychometric properties of the modified BSES-SF among Chinese mothers of preterm infants	Hubei Province, China	153 women < 37 weeks gestation recruited from postpartum ward from two hospitals	Chinese	Postnatal: In-hospital	62.2 ± 16.1	6
Zubaran 2010 [57]	To translate and psychometrically assess a Portuguese version of the BSES-SF	Brazil	89 women living in southern Brazil, recruited from a university teaching hospital	Brazilian Portuguese	Postnatal: Between 2 and 12 weeks	(6.22)	7

^a^The quality assessment score was derived from criteria suggested by Shrestha et al. [14] and Mirza and Jenkins [15]. Scores can range from 0 to 8 with higher scores indicating higher quality

*BSES* Breastfeeding Self-Efficacy Scale*,* *BSES-SF* Breastfeeding Self-Efficacy Scale-Short Form*,* *NR* not reported

The study samples included in the review were diverse in both cultural and demographic characteristics (see Table 1). Six studies assessed the scale in a clinically specific maternal sample including adolescents [39, 41], Canadian Indigenous women [48], black women in the United States [47], and low-income women [25, 40]. In two studies the authors assessed the English version in diverse populations in Australia [26] and the UK [43]. In most studies researchers reported that the scale was used with mothers of term infants. However, three studies reported on the use of the BSES-SF specifically in mothers of ill and preterm infants (< 36 weeks) in Canada [22], Turkey [21], and China [23]. Three studies reported on the use of the BSES-SF for fathers in Canada [19], Malawi [18], and Turkey [20]. Modifications for the BSES-SF scale for mothers of ill and preterm infants included, the addition of four new items, some items were changes to be applicable to mothers of preterm infants, and the item stem was changed from I can to I think I can. Similarly, for the BSES version for fathers, some items were changed to reflect the partner’s experience in assisting the breastfeeding mother.

The scale was translated into 24 different languages, including but not limited to Spanish, Turkish, Swedish, Croatian, Acholi and Langi, Japanese, Portuguese, Italian, Polish, and Malay (Table 2). In 20 studies, there were minor item word modifications. The translation methods were comparable across all studies with most completing back-translation (*n* = 28; 84.8%) to ensure semantic equivalency. However, there were variations noted in the quality of the translation process. Among the studies translated into English (*n* = 33), only 13 (approximately 40%) had at least two qualified personnel doing the forward and back translation with some not specifying if they were independent groups of translators. Eight studies were classified as not applicable regarding translation as they were psychometric studies conducted in English or the tool had been previously translated to English. Pilot testing was conducted in most of the translated studies (*n* = 27; 77.1%) with samples sizes ranging from 5 to 31. A few studies did not report data on pilot testing or translation procedures.

**Table 2:** Translation methods and modifications for all included studies

Author Year	Translated Language	Forward Translation	Back Translation	Pilot-Testing	Other Modifications
**Breastfeeding Self-Efficacy Scale (BSES)**					
Creedy 2003 [26]	NA	NA	NA	NA	None
Dai 2003 [30]	Mandarin Chinese	Yes, by one professional translator	Yes, by two bilingual lay-people	Yes, *n* = 21	Minor wording changes to eight items and removal of one item “I can always keep my baby awake at my breast during a feeding”
Eksioglu 2011 [31]	Turkish	Yes, by five bilingual health professionals	Yes, by a linguist	Yes, *n* = 25	Minor wording changes to three items
Molina Torres 2003 [32]	Spanish	Yes, by three professional translators	Yes, by two bilingual lay-people	Yes, *n* = NR	None
Oriá 2009 [33]	Portuguese	Yes, by two bilingual translators	Yes, two bilingual translators	Yes, *n* = 15 pregnant and *n* = 15 postpartum women	Minor wording changes and changes to Likert response options
**Breastfeeding Self-Efficacy Scale- Short Form (BSES-SF)**					
Amini 2019 [28]	Persian	Yes, by two bilingual translators	Yes, by one bilingual translator	No	None
Asgarian 2020 [34]	Iranian Farsi	Yes, by two bilingual researchers	Yes, by one bilingual translator	Yes, *n* = 10	Minor wording changes to two items
Balaguer- Martinez 2022 [35]	Spanish	NR	NR	NR	NR
Basu 2020 [36]	Hindi	Yes, by one bilingual translator	Yes, by one native translator	Yes, *n* = 10	Sample size included women up to 1 year postpartum; 50% conducted via interview at the request of participants
Boateng 2019 [37]	Acholi and Langi	Yes, by four experts on nutrition, public health, breastfeeding, and medicine	Yes, the same four experts	No	Initially, two items were modified and five items were added to reflect EBF; then up analysis, 7 from the original items and 3 from the added items were dropped, resulting in a 9-items scale
Brandão 2018 [38]	Portuguese	Yes, by two independent bilingual translators	Yes, by two independent bilingual translators	Yes, *n* = 15	Antenatal version (e.g., items stem changes from “I can” to “I think I can”)
Chipojola 2022 [18]	ChiPacewa	Yes, by one bilingual speaker	Yes, by one bilingual speaker	Yes, *n* = 20 fathers	Paternal version minor word changes
Dennis 2011 [39]	NA	NA	NA	NA	Antenatal version: Stem of each question changed from “I can” to “I think I can”
Dennis 2018 [19]	NA	NA	NA	NA	Paternal version: All item stems were changed from “I can always” to “I can always help mom”; the word “my baby” was changed to “our baby”; and finally, an item was changed to acknowledge that the mother breastfeeds
Dodt 2012 [40]	Brazilian Portuguese	NR	NR	NR	NR
Dos Santos 2016 [41]	Brazilian Portuguese	NR	NR	NR	NR
Gerhardsson 2014 [42]	Swedish	Yes, by four breastfeeding experts	Yes, by a professional translator	Yes, *n *= 5	None
Gregory 2008 [43]	NA	NA	NA	NA	None
Handayani 2013 [24]	Indonesia	Yes, by five bilingual translators	Yes, but unclear	No	None
Husin 2017 [27]	Malay	Yes, by two bilingual translators	Yes, by one bilingual translator	Yes, *n* = NR	None
Iliadou 2020 [44]	Greek	NR	NR	NR	NR
Ip 2012 [45]	Hong Kong Chinese	Yes, by 1 of the authors, with reference to Chinese version of the original BSES (Dai and Dennis)	No	Yes, *n *= 12	Minor modifications (unspecified)
Ip 2016 [46]	Mandarin Chinese	NA	NA	Yes, *n* = 10	None – relevant items for the BSES-SF taken from the validated Mandarin version of the full scale
﻿ Küçükoğlu 2023 [20]	Turkish	Yes, by three bilingual translators	Yes, by three bilingual translators	Yes, *n* = 20 fathers	None
Maurer (n.d.) (Maurer, et al.: The breastfeeding self-efficacy scale - short form (BSES): German translation and psychometric assessment, unpublished)	German	Yes, by three independent teams	Yes	Yes, by breastfeeding experts (*n* = 7) and breastfeeding mothers (*n* = 11)	None
McCarter-Spaulding 2010 [47]	NA	NA	NA	NA	None
McQueen 2013 [48]	NA	NA	NA	NA	None
Mituki 2017 [25]	Kiswahili	Yes, by one native speaker	Yes, by one native speaker	NR	Minor wording changes to seven items and the Likert scale phases were modified
Oliver-Roig 2012 [49]	Spanish	Yes, by two bilingual translators	Yes, by two bilingual translators	Yes, *n* = 9	Minor changes, e.g., changed “confidence” in the English version to “self-confidence” in the Spanish version
Otsuka 2008 [50]	Japanese	Yes, separately by first author and a bilingual breastfeeding mother	Yes, by two bilingual translators	Yes, *n* = 11	Two minor wording modifications
Pavicic-Bosnjak 2012 [51]	Croatian	Yes, by two bilingual translators	Yes, by two bilingual lay translators	Yes, *n* = 20	Minor wording modifications on 2 items, and change of Likert-scale from “very confident / not confident” to “strongly agree / strongly disagree”
Petrozzi 2016 [52]	Italian	Yes, by the authors	Yes, by an English-speaker fluent in Italian	Yes, *n* = NR	None
Radwan 2022 [53]	Arabic	Yes, by one bilingual translator	Yes, by two bilingual translators	Yes, *n* = 10	Yes, some questions reworded for clarity
Sandhi 2022 [29]	Bahasa Indonesia	Yes, by one bilingual translator	Yes, by one bilingual translator	Yes, *n* = 10	None
Tokat 2010 [54]	Turkish	Yes, by three bilingual translators	Yes, by one lay bilingual translator	Yes, *n* = 11 pregnant and *n* = 16 postpartum women	Minor change to one item
Tokat 2020 [21]	Turkish	Yes, by three bilingual translators	Yes, by one bilingual translator	Yes, *n* = 12	None
Wheeler 2013 [22]	NA	NA	NA	Yes, *n *= 10	Preterm version: 4 items added to make scale applicable to mothers with ill or pre-term infants
Witten 2020 [55]	Setswana	Yes, by two bilingual translators	Yes, by two bilingual translators	Yes, *n* = 6	Minor wording changes to Likert scale
Wutke 2007 [56]	Polish	Yes, by 5 bilingual graduate students not associated with the project	Yes, by 5 different bilingual graduate students	Yes, *n* = 31	Wording modified for some items to improve clarity
Yang 2020 [23]	Chinese	Yes, by 2 bilingual translators	Yes, by 2 bilingual translators	Yes, *n* = 15	None
Zubaran 2010 [57]	Brazilian Portuguese	Yes, by 2 bilingual investigators	Yes, by the same two investigators	Yes, *n* = 10	None

*BSES* Breastfeeding Self-Efficacy Scale, *BSES-SF* Breastfeeding Self-Efficacy Scale-Short Form, *NA* Not Applicable (Scale was in English or not translated), *NR* Not Reported, *EBF* Exclusive Breastfeeding

### Psychometric assessments of BSES and BSES-SF

#### Reliability

Five studies that evaluated the BSES reported Cronbach's alphas ranging from 0.88 to 0.97 while the remaining 36 studies that assessed the BSES-SF reported Cronbach's alphas ranging from 0.72 to 0.96. Studies validating non-English versions (*n* = 34) had a wider range in Cronbach's alpha’s (0.72—0.95) than the seven studies analyzing English versions (0.88—0.96). The original BSES reported a Cronbach’s alpha of 0.96 [9] and the BSES-SF reported a Cronbach's alpha of 0.94 [10], indicating that the translated and modified versions are comparable. The reliability of all modified tools was further supported by the fact that deletion of any single item did not lead to an increase in Cronbach's alpha of more than 0.10, except one [37]. For studies validating the BSES-SF, the majority of item-total correlations were above the recommended 0.30 criterion.

#### Construct validity

Five studies validated the construct validity of the BSES using factor analysis [26, 30–33], in addition to the original development by Dennis and Faux [9]. The five studies reported a 2-factor solution with eigenvalues ranging from 4.75 to 11.99 and the combined two factors contributing to 29.2% to 57% of the variance. In the original development of the BSES, the 2-factor solution had eigenvalues of 2.75 and 16.87 and the combined two factors contributed to 45.6% of the variance [9]. The 2-factor solution in all cases was consistent with the original BSES factor analysis and congruent with the theorized breastfeeding technique and intrapersonal thoughts subscales [9]. In the studies that completed a factor analysis of the BSES-SF, a single-factor solution was frequently reported, consistent with the original BSES-SF’s unidimensional structure [10].

Construct validity was further assessed in 32 studies using known-groups analysis. It was hypothesized that women who have successfully breastfed in the past would have higher self-efficacy scores than those with no prior breastfeeding experience. All studies (*n* = 18) except two [21, 41] reported significant differences in mean BSES or BSES-SF scores among women who had previously breastfed compared to those with no previous breastfeeding experience (Table 3). Nineteen studies compared mean breastfeeding self-efficacy scores between primiparous and multiparous women, with only half reporting a statistically significant difference based on parity. This finding is consistent with the breastfeeding self-efficacy theory [7] and supports the importance of the information source of performance accomplishment and that previous breastfeeding experience, not parity, is an important indicator of breastfeeding self-efficacy.

**Table 3:** Psychometric properties of the modified versions of the BSES AND BSES-SF for all included studies

Author Year	Cronbach’s alpha	Predictive Validity Assessment		Construct Validity Assessments					
				**Known groups analysis**		**Correlation with related constructs**			
		**Behaviour / factor predicted**	**Predictive (** ***p*** **< 0.05)**	**Comparator groups**	**Construct validity supported**	**Related construct used**	**Time of assessment of related construct**	**Correlation coefficient ® (** ***p*** **< 0.001 unless specified)**	**Construct validity supported?**
**Breastfeeding Self-Efficacy Scale (BSES)**									
Creedy 2003 [26]	Antenatal = .97	Antenatal BSES vs. 1-week BF practices and 16 weeks EBF	Yes	Primiparas vs. multiparas with BF experience	Yes	HHLS	Antenatal	0.73^*^	Yes
	Postnatal = .96	1-week BSES vs. 16 weeks EBF	Yes				4-weeks	0.88^*^	Yes
							16-weeks	0.88^*^	Yes
Dai 2003 [30]	.93	Feeding method, 4 and 8 weeks	Yes	NA	NA	EPDS	4-weeks	-0.35	Yes
							8-weeks	-0.20^**^	Yes
Eksioglu 2011 [31]	1 week = .91 4 weeks = .92	1, 4, 8 weeks BSES vs. EBF	Yes	NA	NA	NA	NA	NA	NA
Milina Torres 2003 [32]	.96	BSES vs. EBF	Yes	Primiparas vs. multiparas with BF experience	Yes	NA	NA	NA	NA
Oriá 2009 [33]	.88	NI	NA	Primiparas vs. multiparas	No	NI	NA	NA	NA
				Satis vs. unsatisfactory BF experiences	Yes				
**Breastfeeding Self-Efficacy Scale- Short Form (BSES-SF)**									
Amini 2019 [28]	.91	NI	NA	Primiparas vs. multiparas	No	EPDS	NR	-0.273	Yes
						PSS-10	NR	-0.068 (NS)	No
Asgarian 2020 [34]	.92	NI	NI	Primiparas vs Multiparas	No	NI	NA	NA	NA
Balaguer-Martinez 2022 [35]	NR	NI	NI	NI	NI	NI	NI	NI	NI
Basu 2020 [36]	.87	NI	NI	Primiparas vs Multiparas	No	EPDS	NR	NR	Yes
						MSPSS	NR	NR	Yes
Boateng 2019 [37]	4-week: Cognitive = .82 Functional = .77 12-week: Cognitive = .85 Functional = .79	4-week: BSES-SF vs. 4, 12, 24 weeks EBF 12-week: BSES-SF vs. 12 and 24 weeks EBF	Yes, but not at 24 weeks Yes	Primiparas vs Multiparas Correct vs. Incorrect BF knowledge	No Yes -Cognitive Subscale No-Functional Subscale	EBFSS	4 and 12 weeks	Cognitive Subscale Informational EBFSS: 0.23; Emotional EBFSS:0.28 BSES-SF Functional Subscale Instrumental EBFSS:0.31; Informational EBFSS: 0.39; Emotional EBFSS:0.47	Yes
						CESD	4 and 12 weeks	BSES-SF Functional Subscale and depression: -.014	Yes
Brandão 2018 [38]	.92	Antenatal BSES-SF vs. 4 weeks EBF	Yes	Primiparas vs. multiparas with BF experience	Yes	STAI	Antenatal and 4, 12, 24 weeks	NI	Yes
		Antenatal BSES-SF vs. 12- and 24-weeks EBF	No	Intention to BF for > 24 weeks vs. < 24 weeks	Yes	EPDS (> 9)	Antenatal and 4, 12, 24 weeks	NI	Yes
Chipojola 2022 [18]	.90	NI	NI	Primiparas vs. multiparas	Yes	WHOQoL-BREF	NR	Psychological well-being = 0.23 Social relations = 0.28 The environment = .30 Physical well-being = .01	Yes, except for physical well-being
Dennis 2011 [39]	Antenatal = .84	Antenatal BSES-SF vs. BF initiation	Yes	Adolescents with prior BF experience vs. those without	Yes (for antenatal but not postnatal)	BAQ	Antenatal	.41	Yes
	Postnatal = .93	Antenatal and postnatal BSES-SF vs. 4-week BF duration and exclusivity	Yes	Decided to BF before vs during pregnancy	Yes (for antenatal but not postnatal)				
Dennis 2018 [19]	Antenatal = .91 Postnatal = .92	Paternal in-hospital BSES–SF vs. 6-weeks and 12-week EBF	No	Paternal in-hospital BSES–SF vs. Maternal in-hospital BSES–SF	Yes	IIFAS	Antenatal & in-hospital	Antenatal = .26 In-hospital = .40	Yes
						Fathers’ perception of breastfeeding importance (designed for study)	In-hospital	.27	Yes
		6-week BSES–SF vs. 12-week EBF	Yes			Involvement in decision making (yes/no)	In-hospital	NR	Yes
						Breastfeeding progress in-hospital (designed for study)	In-hospital	.27	Yes
Dodt 2012 [40]	.74	NI	NI	Primiparas vs. multiparas	No	NI	NI	NI	NI
				Previous BF experience vs. none	Yes				
Dos Santos 2016 [41]	.82	NI	NI	Primiparas vs. multiparas	No	NI	NI	NI	NI
				Previous BF experience vs. none	No				
Gerhardsson 2014 [42]	.91	BSES-SF vs. EBF	Yes	Primiparas vs. multiparas	Yes	NI	NA	NA	NA
				Positive vs. negative BF experiences	Yes				
Gregory 2008 [43]	.90	In-hospital BSES-SF vs. 4-week EBF	Yes	Primiparas vs. multiparas with BF experience	Yes	NI	NA	NA	NA
Handayani 2013 [24]	.77	NI	NA	NI	NA	NI	NA	NA	NA
Husin 2017 [27]	Antenatal: .94 Postnatal: .95	NI	NA	NA	NA	NI	NA	NA	NA
Iliadou 2020 [44]	.93	3-day BSES-SF vs. 6-month EBF	Yes	NI	NA	EPDS	Antenatal	.23 ^*^	Yes
							Postnatal (3 ddp)	-.22 ^*^	Yes
Ip 2012 [45]	.95	BSES-SF and BF duration	Yes	Women with BF experience vs. those without	Yes	NA	NA	NA	NA
		BSES-SF vs. 4-week EBF	Yes						
		BSES-SF vs. 24 weeks EBF	Yes, compared to bottle feeding but not partial BF						
Ip 2016 [46]	.94	BSES-SF at 3 dpp predicted EBF	Yes	NI	NA	NI	NA	NA	NA
Küçükoğlu 2023 [20]	.93	NI	NA	NI	NA	NI	NA	NA	NA
Maurer (n.d.) (Maurer, et al.: The breastfeeding self-efficacy scale - short form (BSES): German translation and psychometric assessment, unpublished)	.88	NI	NA	vs. women with two, three or more children	Yes	NI	NA	NA	NA
McCarter-Spaulding 2010 [47]	.94	In-hospital BSES-SF vs. 4-week BF duration	Yes	Women with BF experience vs. those without	Yes	NSB	1-week and 4-weeks postpartum	1 week = .44 4-weeks = .40	Yes
		In-hospital BSES-SF vs. 4-week EBF	Yes	Intention to BF for > 24 weeks vs. < 24 weeks	Yes				
		In-hospital BSES-SF vs. 24-weeks BF duration	Yes						
McQueen 2013 [48]	.95	In-hospital BSES-SF vs. 4-week EBF	Yes	Primiparas vs. multiparas with BF experience	Yes	EPDS	4 weeks postpartum	NR	Yes
		In-hospital BSES-SF vs. 8-week EBF	Yes						
Mituki 2017 [25]	.91	BSES-SF vs. 6-week EBF	Yes	NI	NA	NI	NA	NA	NA
Oliver-Roig 2012 [49]	.92	In-hospital BSES-SF vs. 3 weeks EBF	Yes	Women with BF experience vs. those without	Yes	GSEI	2 dpp	.5	Yes
						GSES	2 dpp	.24^††^	Yes
						SMSE	2 dpp	.41	Yes
				Women with positive vs. negative BF experiences	Yes	NA	NA	NA	NA
Otsuka 2008 [50]	.95	BSES-SF vs. 4-week EBF	Yes	Primiparas vs. multiparas	Yes	PIM	4 weeks	-.45	Yes
				Women with BF experience vs. those without	Yes				
				Intention to EBF vs. not	Yes				
Pavicic-Bosnjak 2012 [51]	.86	In-hospital BSES-SF vs. 4- and 24-weeks BF duration	Yes	Primiparas vs. multiparas	Yes	SOC	In-hospital	.32	Yes
		In-hospital BSES-SF vs. 4 and 24 weeks EBF	Yes	Multiparas with > 24 weeks vs. ≤ 24 weeks of BF	Yes				
Petrozzi 2016 [52]	.92	In-hospital BSES-SF vs. EBF	Yes	Primiparas vs. multiparas	No	EPDS	In-hospital	− .18^***^	Yes
Radwan 2022 [53]	.95	BSES-SF vs. 24-week EBF	Yes	Primiparas vs. multiparas	Yes	EPDS	NR	-.23	Yes
				Women with BF experience vs. those without	Yes				
Sandhi 2022 [29]	.90	NI	NR	Primiparas vs. multiparas	Yes	EPDS	NR	-.213	Yes
						HADS – depression subscale	NR	-.171 ^*^	Yes
Tokat, 2010 [54]	Antenatal = .87 Postnatal = .86	Antenatal BSES-SF vs. 12-weeks BF duration and EBF	Yes	Women with BF experience vs. those without	Yes	HADS – Anxiety subscale	NR	-.147 ^***^	Yes
		In-hospital BSES-SF vs. 12-weeks BF duration and exclusivity	Yes						
Tokat 2020 [21]	.72	1-week BSES-SF vs. 4-week EBF	Yes	Women with BF experience vs. those without	No	BAI	1 week postpartum	-.219 ^***^	Yes
Wheeler 2013 [22]	.88	1-week BSES-SF vs. 6 weeks BF duration	Yes	Women with BF experience vs. those without	Yes	HHLS	1 week	− .84	Yes
Witten 2020 [55]	.83	BSES-SF vs. 4–8 weeks BF duration and exclusivity	Yes	Primiparous vs. multiparas	Yes	EPDS	NR	-.17	No
Wutke 2007 [56]	.89	In-hospital BSES-SF vs. 8- and 16-weeks BF duration	Yes	Primiparous vs. multiparas	Yes	NI	NA	NA	NA
		In-hospital BSES-SF vs. 8 and 16-week EBF	Yes	Women with BF experience vs. those without	Yes				
Yang 2020 [23]	.973	NI	NA	Primiparous vs. multiparas	Yes	NI	NA	NA	NA
				Women with BF experience vs. those without	Yes				
Zubaran 2010 [57]	.71	BSES-SF vs. EBF	Yes	Primiparous vs. multiparas	No	EPDS	Between 2–12 weeks postpartum	-.41	Yes

*BAQ* Breastfeeding Attitudes Questionnaire, *BAI* Beck Anxiety Inventory, *BSES* Breastfeeding Self-Efficacy Scale, *BSES-SF* Breastfeeding Self-Efficacy Scale-Short Form, *CESD* Center for Epidemiologic Studies Depression Scale, *dpp* days postpartum, *EPDS* Edinburgh Postnatal Depression Scale, *GSEI* Global Self-Efficacy Index, *GSES* General Self-efficacy Scale, *HAS* Hospital Anxiety and Depression Scale, *HHLS* Hill & Humenick Lactation Scale, *MSPSS* Multidimensional Scale of Perceived Social Support, *NA* Not Applicable, *NI* Not Indicated, *NSB* Network Support for Breastfeeding Tool, *PIM* Perception of Insufficient Milk, *PPV* positive predictive value, *NPV* negative predictive value, *PSS* Perceived Stress Scale, *QMIDAT* Questionnaire Measure of Individual Differences in Achieving Tendency, *RSES* Rosenberg Self-esteem Scale, *SMSE* Stress Management Self-Efficacy Scale, *SOC* Sense of Coherence Scale, *week* weeks postpartum

^*^ *p* = < 0.01

^**^ *p* = 0.04

^***^ *p* = < 0.05

^††^ *p* = 0.005

Mean breastfeeding self-efficacy scores were compared across the other known-group variables including: (1) accurate versus inaccurate breastfeeding knowledge [37]; (2) intended breastfeeding duration more than 6 months versus less than 6 months [38, 47]; (3) positive versus negative previous breastfeeding experience [33, 42, 49]; (4) timing of decision to breastfeeding comparing early versus late pregnancy [39]; and, (5) exclusive versus partial breastfeeding (Maurer, et al.: The breastfeeding self-efficacy scale - short form (BSES): German translation and psychometric assessment, unpublished). In all studies, researchers reported significant group differences in mean breastfeeding self-efficacy scores and the scale’s ability to accurately predict group membership. When in-hospital BSES-SF scores were examined between maternal-paternal pairs, a significant correlation (*r* = 0.53, *p* < 0.001) was found [19].

Correlations between breastfeeding self-efficacy scores and other theoretically related constructs were examined in several studies. Constructs for which a hypothesized positive correlation was found included the General Self-Efficacy Scale (GSES) [49]; the Sense of Coherence (SOC) subscales of comprehensibility, manageability and meaningfulness [51]; the Stress Management Self-Efficacy Scale (SMSE) [49]; the Rosenberg Self-Esteem Scale (RSES) [58]; the Hill and Humenick Lactation Scale (HHLS) [22, 26]; the Breastfeeding Attitudes Questionnaire (BAQ) [39]; the Iowa Infant Feeding Attitude Scale (IIFAS) [19]; the Exclusive Breastfeeding Social Support scale (EBFSS) [59]; the Multidimensional Scale of Perceived Social Support [36]; the Network Support for Breastfeeding Tool [47]; the WHO Quality of Life (QoL)-BREF [18]; maternal perceptions of breastfeeding progress [19]; and paternal perceptions of breastfeeding importance [19] (Table 3). Researchers in eleven studies reported a significant negative association between EPDS and BSES scores [28–30, 36, 38, 44, 48, 52, 53, 55, 57]. In particular, women with higher scores on the EPDS had significantly lower BSES scores. Using other measurements of depression, Boateng et al. [37] reported a negative correlation between Centre for Epidemiological Studies Depression Scale (CES-D) [60] and BSES-SF scores and two researchers [29, 54] reported a negative correlation between the HADS depression subscale and BSES-SF scores. All three studies that examined the correlation between anxiety and BSES-SF, researchers found a negative correlation between anxiety and BSES-SF scores, using different measurement tools [21, 29, 38]. Lastly, Otsuka et al. [50] reported a negative correlation between BSES-SF scores and maternal perceptions of insufficient milk supply as measured by the PIM tool. Notably, translated scales also displayed significant construct validity and convergent validity with translated versions of other theoretically related constructs. Thus, evidence of construct and convergent validity in these studies conducted with non-English speaking participants adds to the strength of the psychometric analysis and provides further support for the use of the translated version in the tested population.

#### Predictive validity

The utility of BSES and BSES-SF scores as a means of predicting actual breastfeeding outcomes was assessed in most of the studies, wherein prior mean scores of mothers who breastfed were compared to those who discontinued breastfeeding. In the original BSES validation assessment, mothers still exclusively breastfeeding at six weeks postpartum had significantly higher mean scores (*M* = 173.5, *SD* = 20.9) than those engaging in combination feeding (*M* = 161.9, *SD* = 37.1) or exclusively bottle-feeding (*M* = 145.3, *SD* = 22.4) [9]. Two studies assessed predictive validity of the BSES-SF with regard to breastfeeding initiation outcomes [10, 39] (Table 3). Thirteen studies evaluated the utility of the BSES (*n* = 2) and BSES-SF (*n* = 11) as predictors of breastfeeding duration, and 25 studies reported results on the predictive validity of BSES (*n* = 4) or BSES-SF (*n* = 21) scores as indicators of breastfeeding exclusivity. Statistical significance was reached in all 25 studies except four.

#### Sensitivity and specificity

In two studies [35, 45], researchers evaluated the sensitivity and specificity of the BSES-SF at particular cut-off points using receiver operating characteristic (ROC) curve analysis. Sensitivity provides an estimate of the tool’s accuracy in identifying mothers at risk of premature breastfeeding cessation (i.e., the proportion of the sample that discontinued breastfeeding prior to the time period in question and scored below the identified cut-off). In contrast, specificity refers to the proportion of the sample that did not discontinue breastfeeding before the assessed time period and scored above the identified cut-off. In the Hong Kong Chinese version of the BSES-SF, Ip et al. [45] identified the optimal cut-off for predicting early breastfeeding cessation (before 6 months) to be a score of 45.5 out of 70. Sensitivity at this cut-off score was 77%, and specificity was 73%. The negative predictive value of the scale indicated that 92% of mothers who scored below 45.5 discontinued breastfeeding prior to 6 months postpartum. In the Spanish version of the BSES-SF, Balaguer-Martinez et al. [35] found that the area under the curve was above the threshold for good predictive power for mothers who were exclusively breastfeeding at 1 and 2 months. To achieve 80% sensitivity the BSES-SF cut-off score was 59 at 1 month and 58 at 2 months. These findings demonstrate the utility of the BSES-SF as a tool to identify mothers at risk of prematurely discontinuing breastfeeding.

## Discussion

In this systematic review, an evaluation of the reliability and validity of the BSES and BSES-SF scales in multiple languages, as well as their psychometric assessments in specific perinatal populations, including fathers [18–20] and parents with an ill or preterm infant [21–23], was conducted to appraise their effectiveness in identifying women and partners at risk of poor breastfeeding outcomes. This review is the first to evaluate the psychometrics of translated and adapted versions of the BSES and BSES-SF among demographically and culturally diverse populations. Additionally, this is the first review to assess the rigour and quality of the studies that have adapted and applied the BSES or BSES-SF for measuring breastfeeding self-efficacy. The BSES and BSES-SF have been psychometrically tested in 41 studies and translated into 26 languages other than English. Psychometric properties of the BSES and BSES-SF reported in the included studies were comparable to the original studies completed by Dennis [9, 10] indicating the utility of the instrument as an adaptable and reliable tool for measuring breastfeeding self-efficacy in diverse populations and settings. However, our review also found that some studies had deficiencies in their translation and cultural adaptation processes. Thus, while the BSES and BSES-SF appear to have sound psychometric properties across studies, caution in the interpretation of the findings should be considered as cultural aspects may not have been captured by the instruments [14]. Future studies should use established methodological approaches [14, 61] for translating and adapting the BSES-SF for use in cross-cultural research to ensure important cultural nuances are included in translated and culturally adapted tools.

Reliability was indicated with all Cronbach alphas coefficients exceeding the recommended 0.70 for established instruments (Nunnally and Bernstein: Psychometric theory, unpublished). There was variability between studies with some translated studies having wider Cronbach alpha coefficients than the non-translated studies. This could be due to the sample size, slight modifications made during the translation or adaptation process, cultural nuances that might not be captured in the translated version or varying sample characteristics of the included studies. We anticipated some variability and do not believe that this is an appreciable difference as all studies exceeded the recommendation. As such, this does not impact the overall reliability of the BSES-SF to be used in diverse samples effectively to guide interventions.

The modified versions of the BSES and the short form were shown to be conceptually valid with the majority of the studies reporting expected correlations with other widely used and theoretically related constructs such as the depression, anxiety, general self-efficacy, breastfeeding attitude, and sense of coherence. Moreover, considering the main results from across all studies, the BSES and BSES-SF and their adapted versions demonstrated significant predictive validity at various time points in the postpartum period. This finding supports the notion that even after translation or modification of these scales, they remain useful tools for identifying mothers at risk of negative breastfeeding outcomes in terms of all three breastfeeding parameters: initiation, duration, and exclusivity. This has significant implications for applying the BSES and BSES-SF in future studies across various cultural and demographic contexts.

Conceptual equivalence was another key indicator in measuring the comparability of the original English BSES and BSES-SF with its adapted, translated versions. While most of the studies identified used forward and back-translation, it is important to note that some studies used lower quality translation processes with only one individual performing the translation and in other studies limited details were provided. Back-translation to English may have been influenced by translators possessing knowledge of the original English version of the scale, especially if forward and back-translations were conducted by the same group. This method may result in conceptual disparities between the original English BSES and its short form and their translated versions. Thus, it is important that rigorous translation and adaptation processes be used to enhance the validity and reliability of the instrument (e.g., BSES-SF) for use among individuals of diverse cultures and languages [61].

While the BSES and BSES-SF were widely used globally, we found that middle to higher income countries have predominantly adapted and validated the tool. This is important to acknowledge as cultural and demographic influences may have led to higher breastfeeding self-efficacy in some studies. In countries with higher rates of breastfeeding, women are typically more often exposed to the primary sources of self-efficacy (e.g., antecedents) [7] such as vicarious experience (seeing others breastfeed) and verbal persuasion (receiving positive reinforcement). Furthermore, women may have received more assistance (e.g., education and support) [5] with breastfeeding in some settings thereby enhancing others sources of information such as performance accomplishment.

In many low and middle-income countries, there is a growing burden of breastfeeding attrition and increased reliance on formula use [62]. Major contributing factors to this trend are the influence of the private sector in promoting formula as an alternative to breastfeeding, lack of access to health care professionals and support from caregivers, limited education, and poor awareness stemming from broader political and economic disadvantages [4, 61, 63]. These contributing factors highlight the need for continued analyses of the factors associated with low breastfeeding initiation, duration and exclusivity, and underline the importance of developing reliable and valid instruments for identifying mothers most at risk of developing suboptimal breastfeeding practices. The psychometrics of such tools should be assessed in a wide range of languages, demographic contexts, and cultural settings. While our review found that the BSES and BSES-SF are adaptable, reliable, and validated tools globally, the benefits of the tool have not been tested and evaluated in resources poor settings.

Overall, these findings have important implications globally for clinical practice. The BSES and BSES-SF appear to be reliable and valid tools that may be used to assess mothers’ breastfeeding self-efficacy and plan interventions that are based on maternal need [7]. While our review included studies that assessed breastfeeding self-efficacy at various time periods, the antenatal period and the early postpartum period, soon after delivery, have the most clinical utility for assessing breastfeeding self-efficacy. As the main purpose in administering the BSES-SF is to identify women who may be at risk for early breastfeeding discontinuation, assessment of breastfeeding self-efficacy later in the postpartum period is not as clinically relevant as infant feeding has typically been established.

The total BSES score (14 – 70) may be used to identify mothers with low breastfeeding self-efficacy who may be at risk for negative breastfeeding outcomes (e.g., initiation, duration and exclusivity) and may benefit from additional supportive interventions [64]. Conversely, mothers who have high breastfeeding self-efficacy may be recognized as being more likely to succeed with breastfeeding; however, additional assistance may still be required, particularly when experiencing breastfeeding difficulties [7]. It is noteworthy that we do not recommend a specific cut-off score (e.g., total score) to delineate high versus low breastfeeding self-efficacy as breastfeeding self-efficacy scores can be culturally specific and vary. The single item BSES scores (1 – 5) can also be used to assess maternal perceptions of self-efficacy regarding specific components of breastfeeding (e.g., determine the baby is getting enough, proper latch, exclusive breastfeeding, etc.). Individual items scores (1 – 5) can be used to identify perceptions of low self-efficacy (item score ≤ 3) and items where the mother feels efficacious (item score ≥ 4) so that support specific to individual maternal needs can be provided [64]. The BSES has also been utilized to develop and/or evaluate the efficacy of various types of supportive interventions [5, 6]. Finally, the BSES assessment may provide health care professionals with a better of understanding of where mothers lack breastfeeding confidence and why they may be unsuccessful despite additional support [7].

## Limitations

Our systematic review has several limitations. First, we found that only two studies included sensitivity and specificity data and reported negative and positive predictive values. Hence, we were not able to conduct a comprehensive analysis of these parameters. Second, the quality of the translation and cultural adaptation among several studies was lacking. Some studies also had missing data (e.g., not reported) affecting the assessment of reliability and validity. Finally, while most studies in this review employed very similar validation methods, the timing of assessment varied among studies as did the characteristics and geographic location of the study participants. The heterogeneity of the samples can make comparison across studies difficult; however, the consistency of the findings between studies also suggests the versatility of the tool among diverse groups.

## Conclusion

Breastfeeding is a practice that is approached and perceived differently among different cultures, and premature discontinuation of breastfeeding is a global public health concern. Continued efforts are needed in the cross-cultural adaptation of the BSES and BSES-SF to effectively serve diverse populations and provide contextually appropriate measures of breastfeeding self-efficacy. We recommend that future studies validating translated or adapted versions of the BSES and BSES-SF adopt more systematic approaches to empirical validation, cultural adaption, and translation of the scales that are consistent with those used in the original analysis of the psychometric properties of the BSES and BSES-SF. Considering the extent to which the BSES and BSES-SF take into account the needs and perceptions of non-English-speaking mothers’ self-efficacy, further efforts should be made to translate the BSES into other languages.

### Supplementary Information

**Additional file 1:Table S1. **Search strategy.

**Additional file 2:Table S2. **Study quality assessment for all included studies (and the original scales).

## Data Availability

Data sharing is not applicable to this article as no datasets were generated or analysed during the current study.

## Abbreviations

BAQ: Breastfeeding Attitudes Questionnaire
BAI: Beck Anxiety Inventory
BSES: Breastfeeding Self-Efficacy Scale
BSES-SF: Breastfeeding Self-Efficacy Scale-Short Form
CESD: Center for Epidemiologic Studies Depression Scale
Dpp: Days postpartum
EPDS: Edinburgh Postnatal Depression Scale
GSEI: Global Self-Efficacy Index
GSES: General Self-efficacy Scale
HAS: Hospital Anxiety and Depression Scale
HHLS: Hill & Humenick Lactation Scale
MSPSS: Multidimensional scale of perceived social support
NA: Not Applicable
NI: Not Indicated
NSB: Network Support for Breastfeeding Tool
PIM: Perception of Insufficient Milk
PPV: Positive predictive value
NPV: Negative predictive value
PSS: Perceived Stress Scale
QMIDAT: Questionnaire measure of individual differences in achieving tendency
RSES: Rosenberg Self-esteem Scale
SMSE: Stress Management Self-Efficacy Scale
SOC: Sense of Coherence Scale
