# Supplementary material for: Psychometrics of the breastfeeding self-efficacy scale and short form: a systematic review
Source: BMC Public Health. 2024 Feb 29;24:637. doi: 10.1186/s12889-024-17805-6 (PMC10903029; doi:10.1186/s12889-024-17805-6)
Supplement: Supplementary file 1 — Additional file 1:Table S1. Search strategy. [file 12889_2024_17805_MOESM1_ESM.docx]

**Table S1**

*Search Strategy*

| Database | Search Strategy |
| --- | --- |
| EMBASE | ('breastfeeding self-efficacy scale':ti,ab,kw OR 'breastfeeding':ti,ab,kw OR 'breastfeeding'/exp OR 'self-efficacy'/exp OR 'self-efficacy':ti,ab,kw) AND ('cultural adaptation':ti,ab,kw OR 'culture':ti,ab,kw OR 'cultural adaptation'/exp) AND ('psychometric':ti,ab,kw OR 'validity':ti,ab,kw OR reliability:ti,ab,kw OR 'cultural adaptation'/exp OR 'validity'/exp OR 'reliability'/exp) |
| MEDLINE | (breastfeeding self-efficacy scale[Title/Abstract]) OR breastfeeding[MeSH Terms]) OR self-efficacy[MeSH Terms]) OR self-efficacy[Title/Abstract]) AND (cultural adaptation[Title/Abstract]) OR culture[Title/Abstract]) OR cultural adaptation[MeSH Terms]) AND (lib) AND full text[sb] AND Humans[Mesh]) Sort by: Best Match Filters: Full text; Humans; English |
| PsycINFO | ("breastfeeding self-efficacy scale".ab. or "breastfeeding self-efficacy scale".ti. or "breastfeeding".ab. or "breastfeeding".ti. or "self-efficacy".ab. or "self-efficacy".ti. or breastfeed.mh. or self-efficacy.mh.) and ("cultural adaptation".ab. or "cultural adaptation".ti. or culture.ab. or culture.ti. or "cultural adaptation".mh.) and ("psychometric".ab. or "psychometric".ti. or "validity".ab. or "validity".ti. or "reliability".ab. or "reliability".ti. "psychometric".mh. or "validity".mh. or "reliability".mh.) limit to (full text and human) |
