# Supplementary material for: Psychometrics of the breastfeeding self-efficacy scale and short form: a systematic review
Source: BMC Public Health. 2024 Feb 29;24:637. doi: 10.1186/s12889-024-17805-6 (PMC10903029; doi:10.1186/s12889-024-17805-6)
Supplement: Supplementary file 2 — Additional file 2:Table S2. Study quality assessment for all included studies (and the original scales). [file 12889_2024_17805_MOESM2_ESM.doc]

**Table S2**

*Study Quality Assessment for All Included Studies (and the Original Scales)*

| **Author Year** | **Clear Study Aim** | **Sample Size Justified** | **Sample Representative of General Population ^A^** | **Inclusion and Exclusion Criteria Explained** | **Response Rate Reported** | **Description of Demographic Data** | **Appropriate Statistical Analyses** | **Informed Consent From Participants Obtained** | ***Total Score** |
| --- | --- | --- | --- | --- | --- | --- | --- | --- | --- |
| **Breastfeeding Self-Efficacy Scale (BSES)** | | | | | | | | | |
| Creedy 2003 (27) | 1 | 1 | 1 | 1 | 1 | 1 | 1 | 1 | 8 |
| Dai 2003 (38) | 1 | 0 | 0 | 1 | 1 | 1 | 1 | 1 | 6 |
| Eksioglu 2011 (39) | 1 | 0 | 0 | 1 | 1 | 1 | 1 | 1 | 6 |
| Molina Torres 2003 (40) | 1 | 0 | 0 | 1 | 1 | 1 | 1 | 1 | 6 |
| Oriá 2009 (41) | 1 | 0 | 0 | 1 | 0 | 1 | 1 | 1 | 5 |
| **Breastfeeding Self-Efficacy Scale- Short Form (BSES-SF)** | | | | | | | | | |
| Amini 2019 (29) | 1 | 1 | 0 | 0 | 1 | 1 | 1 | 1 | 6 |
| Asgarian 2020 (64) | 1 | 1 | 1 | 1 | 1 | 1 | 1 | 1 | 8 |
| Balaguer-Martinez 2022 (59) | 1 | 1 | 1 | 1 | 0 | 1 | 1 | 1 | 7 |
| Basu 2020 (49) | 1 | 0 | 1 | 1 | 1 | 1 | 1 | 1 | 7 |
| Boateng 2019 (37) | 1 | 0 | 0 | 1 | 1 | 1 | 1 | 1 | 6 |
| Brandão 2018 (42) | 1 | 0 | 0 | 1 | 1 | 1 | 1 | 1 | 6 |
| Chipojola 2022 (18) | 1 | 1 | 0 | 1 | 1 | 1 | 1 | 1 | 7 |
| Dennis 2011 (31) | 1 | 0 | 0 | 1 | 1 | 1 | 1 | 1 | 6 |
| Dennis 2018 (19) | 1 | 0 | 0 | 1 | 1 | 1 | 1 | 1 | 6 |
| Dodt, 2012 (35) | 1 | 0 | 1 | 1 | 1 | 1 | 1 | 0 | 6 |
| Dos Santos, 2016 (32) | 1 | 0 | 1 | 1 | 1 | 1 | 1 | 1 | 7 |
| Gerhardsson 2014 (44) | 1 | 1 | 0 | 1 | 1 | 1 | 1 | 1 | 7 |
| Gregory 2008 (36) | 1 | 0 | 1 | 1 | 1 | 1 | 1 | 1 | 7 |
| Handayani 2013 (25) | 1 | 0 | 1 | 1 | 0 | 1 | 1 | 1 | 6 |
| Husin 2017 (28) | 1 | 1 | 0 | 1 | 1 | 1 | 1 | 1 | 7 |
| Iliadou 2020 (50) | 1 | 1 | 1 | 1 | 1 | 1 | 1 | 1 | 8 |
| Ip 2012 (58) | 1 | 0 | 1 | 1 | 1 | 1 | 1 | 1 | 7 |
| Ip 2016 (65) | 1 | 0 | 0 | 1 | 1 | 1 | 1 | 1 | 6 |
| ﻿Küçükoğlu 2023 (20) | 1 | 0 | 1 | 1 | 0 | 1 | 1 | 1 | 6 |
| Maurer (n.d.) (45) | 1 | 0 | 1 | 1 | 1 | 1 | 1 | 1 | 7 |
| McCarter-Spaulding 2010 (34) | 1 | 1 | 1 | 1 | 0 | 1 | 1 | 1 | 7 |
| McQueen 2013 (33) | 1 | 1 | 1 | 1 | 1 | 1 | 1 | 1 | 8 |
| Mituki 2017 (26) | 1 | 0 | 1 | 1 | 0 | 1 | 1 | 1 | 6 |
| Oliver-Roig 2012 (43) | 1 | 0 | 0 | 1 | 1 | 1 | 1 | 1 | 6 |
| Otsuka 2008 (57) | 1 | 0 | 0 | 1 | 1 | 1 | 1 | 1 | 6 |
| Pavicic-Bosnjak 2012 (46) | 1 | 0 | 0 | 1 | 1 | 1 | 1 | 1 | 6 |
| Petrozzi 2016 (51) | 1 | 0 | 0 | 1 | 0 | 1 | 1 | 1 | 4 |
| Radwan 2022 (54) | 1 | 1 | 1 | 1 | 1 | 1 | 1 | 1 | 8 |
| Sandhi 2022 (30) | 1 | 0 | 1 | 1 | 0 | 1 | 1 | 1 | 6 |
| Tokat, 2010 (56) | 1 | 1 | 1 | 1 | 1 | 1 | 1 | 1 | 8 |
| Tokat 2020 (21) | 1 | 0 | 1 | 1 | 1 | 1 | 1 | 1 | 7 |
| Wheeler 2013 (22) | 1 | 0 | 0 | 1 | 0 | 1 | 1 | 1 | 5 |
| Witten, 2020 (52) | 1 | 1 | 1 | 1 | 1 | 1 | 1 | 1 | 8 |
| Wutke 2007 (66) | 1 | 1 | 1 | 1 | 1 | 1 | 1 | 1 | 8 |
| Yang 2020 (23) | 1 | 0 | 1 | 1 | 0 | 1 | 1 | 1 | 6 |
| Zubaran 2010 (53) | 1 | 1 | 0 | 1 | 1 | 1 | 1 | 1 | 7 |
| *Note.* ^A^As some studies purposely validated the BSES among a culturally or demographically specific group, representativeness here refers to generalizability to other women with these characteristics of interest, rather than women more generally. BSES = Breastfeeding Self-Efficacy Scale BSES-SF = Breastfeeding Self-Efficacy Scale-Short Form *The quality assessment score was derived from criteria suggested by Shrestha et al. (14) and Mirza and Jenkins (15). Scores can range from 0 to 8 with higher scores indicating higher quality. | | | | | | | | | |
